# Dental Tissue — New Source for Stem Cells

**DOI:** 10.1100/tsw.2009.125

**Published:** 2009-10-14

**Authors:** Vladimir Petrovic, Vladisav Stefanovic

**Affiliations:** University of Nis School of Medicine, 18000 Nis, Serbia

**Keywords:** dental stem cells, multilineage differentiation, odontogenesis, neural disease, tooth tissue engineering

## Abstract

Stem cells have been isolated from many tissues and organs, including dental tissue. Five types of dental stem cells have been established: dental pulp stem cells, stem cells from exfoliated deciduous teeth, stem cells from apical papilla, periodontal ligament stem cells, and dental follicle progenitor cells. The main characteristics of dental stem cells are their potential for multilineage differentiation and self-renewal capacity. Dental stem cells can differentiate into odontoblasts, adipocytes, neuronal-like cells, glial cells, osteoblasts, chondrocytes, melanocytes, myotubes, and endothelial cells. Possible application of these cells in various fields of medicine makes them good candidates for future research as a new, powerful tool for therapy. Although the possible use of these cells in therapeutic purposes and tooth tissue engineering is still in the beginning stages, the results are promising. The efforts made in the research of dental stem cells have clarified many mechanisms underlying the biological processes in which these cells are involved. This review will focus on the new findings in the field of dental stem cell research and on their potential use in the therapy of various disorders.

## INTRODUCTION

The discovery of progenitor/stem cells was a giant breakthrough in medicine and opened the door for a whole new era of experimentation, with their potential use in the therapy of various disorders. Stem cells are defined as clonogenic cells capable of both self-renewal and multilineage differentiation[1]. According to the ability and potency to differentiate into different cellular types, three types of stem cells have been established: (1) totipotent stem cells – each cell has the capability of developing into an entire organism, (2) pluripotent stem cells – embryonic stem cells that were grown *in vivo* under induced conditions and are capable of differentiating into all types of tissue, and (3) multipotent stem cells – postnatal stem cells or adult stem cells with the capability of multilineage differentiation[2].

Postnatal stem cells have been isolated from various organs, including dental tissue[3]. So far, five types of dental stem cells have been identified: dental pulp stem cells (DPSC), stem cells from exfoliated deciduous teeth (SHED), stem cells from apical papilla (SCAP), periodontal ligament stem cells (PDLSC), and dental follicle progenitor cells (DFPC)[4,5,6,7,8]. Dental stem cells belong to the multipotent stem cell population[9].

This paper will focus on the characteristics of dental stem cells, as well as on their potential use for therapeutic purposes.

## CHARACTERISTICS OF DENTAL TISSUE STEM CELLS

### Dental Pulp Stem Cells

The presence of stem cells in dental pulp was proposed by Fitzgerald et al.[10]. They reported the presence of the fibroblast-like cells capable of differentiation into odontoblasts, after the injury or trauma that led to the death of the mature odontoblasts. However, the identification and isolation of DPSC in the adult dental pulp was first reported by Gronthos and coworkers in 2000[4].

The DPSC represent less than 1% of the total cell population present in dental pulp[11]. It is believed that these cells reside in the various regions inside the dental pulp. In adult dental tissue, the stem cell niches are usually quiescent and become activated only after injury[12]. There is some evidence for a role of notch signaling in DPSC differentiation and proliferation[14,15], and labeling studies have found that the odontoblast-subodontoblast layer expresses higher levels of Notch 1, while the pulp proper predominantly expresses Notch 2, and DPSC in perivascular regions show increases in Notch 1 and 3[11,13,14,15].

Previous attempts to isolate and characterize clonal stem cell lines in dental pulp reported the presence of STRO-1–positive cells, with high colony-forming efficiency. These cells were positive for vascular cell markers CD146 and pericyte antigen 3G5[5,16]. The stem cell clone from dental pulp that expressed CD34 and the putative stem cell proto-oncogene marker c-kit, with the capability of differentiation toward stromal lineages, especially osteoblasts, was recently isolated[17,18]. A side cell population, which fails to incorporate the DNA binding dye Hoechst 33342 with stem cell characteristics, has also been isolated from dental pulp[19].

However, the later studies focusing on DPSC odontogenic and proliferative potential reported different results after the implantation of the single-cell colonies of DPSC isolated from the adult dental pulp[3,20]. These differences could be explained by the findings that there is more than one stem cell population in the dental pulp. So far, at least two different stem cell populations were identified, one originating from the neural crest (the derivate of ectomesenchyme) and the other of mesenchymal origin[12].

Both clonal stem stell populations show expression of STRO-1 and classical adult stem cell markers, as well as of Msx-1 and CD31. Also, all cells were positive for vimentin, which proves their mesenchymal origin. Cells of mesenchymal origin were strongly positive for $\beta$1-integrin, which interacts with fibronectin (FBN) of the extracellular matrix in dental pulp, and these cells are referred as +FBN, while the cells originating from the neural crest showed high expression of neural crest–associated low-affinity nerve growth factor receptor (+LANGFR cells). Compared to +LANGFR cells, +FBN cells did not show the expression of endothelial cell markers CD105 and Notch2, and it is thought that these cells reside in the stem cell niches outside the perivascular regions[9,12]. It is thought that only STRO-1–positive cells have the capability of differentiating into odontoblastic lineage[9].

The results are, however, inconclusive. The work in isolation of different stem cell populations in dental pulp is still in the beginning stages. The difficulty lies in the lack of specific cell surface markers.

DPSC and bone marrow–derived stem cells (BMDSC) show similar gene expression for more than 4000 genes, with only a few differentially expressed genes. A few differentially expressed genes, including collagen type XVIII $\alpha$1, insulin-like growth factor-2 (IGF-2), discordin domain tyrosine kinase 2, NAD(P)H menadione oxidoreductase, homolog 2 of *Drosophila* large disk, and cyclin-dependent kinase 6, were highly expressed in DPSC, whereas insulin-like growth factor binding protein-7 (IGFBP-7) and collagen type I $\alpha$2 were more highly expressed in BMDSC[21]. DPSC show a 30% higher proliferation rate than BMDSC under the same culture conditions, probably because of strong expression of cyclin-dependent kinase 6, a cell cycle activator[21].

The studies showed that DPSC can be cryopreserved and retain their multipotential differentiation ability[22]. These cells, under the right conditions, have better immunoregulatory capacity that abolishes T-cell alloreactivity than BMDSC. However, DPSC may not elicit humoral immune responses and may thus be immunoprivileged[23,24].

The proliferation and differentiation of DPSC usually occur after infection or injury of the dental pulp during bacterial infection. The studies showed that local dentin pulp inflammation interfered with odontoblast differentiation and dentin repair. It was reported that lipopolysaccharide (LPS), a major component of the outer membrane of bacteria, and tumor necrosis factor (TNF), abundantly present in the inflamed dental pulp tissue, activate NF-κβ signaling pathway in DPSC. NF-κβ regulates the expression of a variety of inflammatory cytokines, including TNF, IL-1, IL-8, and IL-6. These results suggest that DPSC also have a role in inhibiting oral and dental inflammation[25].

## Stem Cells from Exfoliated Deciduous Teeth

SHED were isolated from dental pulp derived from exfoliated deciduous teeth. Compared to DPSC, SHED show a higher proliferation rate, increased cell-population doublings, sphere-like cell-cluster formation, osteoinductive capacity *in vivo*, and failure to reconstitute a complete dentin pulp–like complex. SHED were found to express the cell surface molecules STRO-1 and CD146, two early mesenchymal stem cell markers previously found to be present in BMDSC and DPSC. STRO-1– and CD146-positive cells were found to be located around blood vessels of the pulp, implying that SHED possibly originate from a perivascular microenvironment. Cultured SHED also express stromal- and vascular-related markers: antialkaline phosphatase (ALP), matrix extracellular phosphoglycoprotein (MEPE), basic fibroblast growth factor (bFGF), and endostatin[7].

These cells have the ability to differentiate into odontoblast-like cells and the regenerated dentin shows immunoreactivity to dentin-specific dentin sialophosphoprotein (DSPP) antibody. Also, SHED are capable of inducing recipient murine cells to differentiate into bone-forming cells. SHED expressed a variety of neural cell markers, including nestin, βIII-tubulin, glutamic acid decarboxylase (GAD), neuronal nuclei (NeuN), 2,3-cyclic nucleotide-3-phosphodiesterase (CNPase), glial fibrillary acidic protein (GFAP), and neurofilament M (NFM). They have the ability to transform into neuronal-like cells, glial cells, but also into adipocytes and chondrocytes[7,26].

SHED probably represent a population of more immature multipotent stem cells than DPSC.

## Stem Cells from Apical Papilla

SCAP appear to be a different population of stem cells from DPSC. In developing teeth, root formation starts as the epithelial cells from the cervical loop proliferate apically, and influence the differentiation of odontoblasts from undifferentiated mesenchymal cells and cementoblasts from follicle mesenchyme. It has been known that the dental papilla contributes to tooth formation and eventually converts to pulp tissue[27,28,29]. As the root continues to develop after the bell stage, the location of the dental papilla becomes apical to the pulp tissue.

SCAP show a two- to threefold higher proliferation rate than do DPSC after the BrdU incorporation. SCAP, similar to DPSC, are more committed to osteo/dentinogenicity. Despite that SCAP express many osteo/dentinogenic markers following *ex vivo* expansion, they express lower levels of DSPP, MEPE, transforming growth factor (TGF)βRII, fibroblast growth factor (FGF)R3, and CD146 than do DPSC. The *in situ* immunohistochemical staining of various osteo/dentiogenic markers revealed that only odontoblasts in the pulp express these genes, not in the apical papilla. Their adipogenic aspect of the multipotentiality was also confirmed[30].

SCAP exhibit a heterogeneous nature by showing (1) coexpression of STRO-1 with a variety of osteo/dentinogenic markers and (2) a low percentage of STRO-1–positive cells, while a high percentage of cells positive with osteo/dentinogenic markers in cultures. In addition, SCAP show a positive staining for several neural markers, including βIII-tubulin, GAD, NeuN, nestin, GFAP, NFM, neuron-specific enolase (NSE), and CNPase. It is possible that SCAP are derived from neural crest cells or at least associated with neural crest cells, analogous to dental stem cells such as DPSC and SHED[30,31].

The discovery of SCAP may also explain a clinical phenomenon, apexogenesis, that can occur in infected immature permanent teeth with periradicular periodontitis or abscess[32]. It is likely that stem cells residing in the apical papilla survived the infection due to their proximity to the periapical tissues. Therefore, after endodontic disinfection, under the influence of the survived Hertwig's epithelial root sheath (HERS), these cells give rise to primary odontoblasts to complete the root formation[30,31].

## Periodontal Ligament Stem Cells

The periodontal ligament (PDL) is a specialized connective tissue derived from the dental follicle and originates from neural crest cells. The PDL connects the cementum to the alveolar bone, and functions primarily to support the tooth in the alveolar socket[33,34].

A recent report identified stem cells in human PDL and found that PDLSC implanted into nude mice generated cementum/PDL-like structures that resemble the native PDL as a thin layer of cementum that interfaced with dense collagen fibers, similar to Sharpey's fibers[3]. These cells can also differentiate into adipocytes, odontoblasts, myotubes, NFM-positive neuron-like cells, GFAP-positive astrocyte-like cells, and CNPase-positive oligodendrocyte-like cells[7,35,36].

PDLSC and DPSC both showed similar characteristics as compared to BMDSC. All cell types were strongly positive for CD44, CD90 (cell surface markers associated with stromal cells), CD105, and CD166 (cell surface markers associated with stromal cells and endothelial cells), but negative for CD40, CD80, and CD86 (cell surface markers of hematopoietic cells)[37].

PDLSC and DPSC expressed HLA-ABC (MHC class I antigen) similar to BMDSC, while HLA-DR (MHC class II antigen) expression was not detected in these cell populations[37].

*Ex vivo* expanded PDLSC and DPSC possess immunosuppressive properties and mediate suppression mainly by soluble factors, partly induced by IFN-γ produced by activated peripheral blood mononuclear cells (PBMNC). Inhibitory factors include indoleamine 2, 3-dioxygenase (IDO) that is dependent on IFN-γ produced by PBMNC, TGF-β1 and hepatocyte growth factor (HGF) that are independent on IFN-γ[37].

The possibility of obtaining stem cells from cryopreserved adult human PDL[38] indicates that samples from tissue banks could be viable for future applications.

## Dental Follicle Progenitor Cells

DFPC are localized in the dental follicle, a mesenchymal tissue that surrounds the tooth germ and can be easily isolated after wisdom tooth extraction[8,39]. However, these cells are available only from patients during wisdom tooth eruption, usually between 15 and 28 years of age[40].

Human DFPC have the ability to differentiate toward alveolar osteoblasts, PDL fibroblasts, cementoblasts, adipocytes, and neuron-like cells[8,39,40,41]. Long-term cultures of DFPC with dexamethasone produced compact calcified nodules or appeared as plain membrane structures of different dimensions consisting of a connective tissue–like matrix encapsulated by a mesothelium-like cellular structure[8]. DFPC differentially express osteocalcin and bone sialoprotein after transplantation in immunocompromised mice, but without any sign of cementum or bone formation. DFPC are fibroblast-like, colony-forming, and plastic-adherent cells[8]. These cells show the expression of putative stem cell markers Notch-1, nestin, and STRO-1[8,39,42]. Compared to BMDSC, DFPC express higher amounts of IGF-2 transcripts[8]. Also, these cells are positive for vimentin, which is a typical marker for mesenchymal cells[39].

## DENTINOGENESIS

Odontoblasts are postmitotic, terminally differentiated cells whose main role is the production of primary dentin. Following the primary dentinogenesis, these cells remain functional and are able to produce the physiologically secondary dentin and the tertiary atubular reparative dentin as a response to mild environmental stimuli. However, any greater injury or trauma leads to their death and, consequently, to the loss of the reparative potential of the dental pulp[43]. Therefore, one of the biggest problems in dentistry today is how to restore diseased dental tissue and to save the vitality of the tooth.

It has been reported that bone sialoprotein and dentin matrix components are able to stimulate reparative dentin formation, and that growth factors such as TGF-β and bone morphogenetic proteins (BMP) are capable of stimulating secretion of extracellular matrix by odontoblasts[44,45,46,47]. Also, *in vivo* gene therapy with Bmp11/Gdf11 by ultrasound-mediated gene delivery stimulated reparative dentin formation on the amputated dental pulp[48]. Recently, it was shown that TNF-α stimulates differentiation of dentin pulp cells toward an odontoblastic phenotype via regulation of mitogen activated protein kinase p38[49]. However, the restoration of damaged dentin is still limited to conventional treatments, such as fillings of dental pulp with calcium hydroxide and crown restorations[50,51]. The dental stem cells and their ability to differentiate into functional odontoblasts could be the answer to the problem.

The possible role for DPSC in regeneration is demonstrated by their *in vitro* differentiation into odontoblast-like cells and deposition of mineralized deposits after treatment with dentin matrix extracts in association with a mineralization supplement of B-glycerophosphate and ascorbic acid[52]. DPSC/HE/TCP implants (hydroxyapatite/tricalcium phosphate ceramic powder) were able to induce the production of dentin with organized dentin tubules, unlike DPSC/dentin implants. It has been shown that DPSC and SHED express DSPP in xenogenic transplants, and this expression is not present in bone formed by bone marrow stromal cells in similar transplants, suggesting that the clonogenic dental pulp–derived cells represent an undifferentiated preodontogenic phenotype *in vitro*[53].

The use of DPSC in everyday clinical work is still not possible because some aspects of such a therapeutic approach need to be explored further. The isolation and characterization of stem cells from the vital pulp is neither feasible, nor practical, in clinical work. Tissue engineering with the triad comprising dental pulp progenitor/stem cells, morphogens, and scaffolds may provide a useful alternative method for pulp-capping and root canal treatment[48]. Such approaches to clinical therapies, however, require close control or regulation of the regenerative events. Uncontrolled regeneration would consequently lead to the pulp obliteration and loss of tooth vitality[54].

The studies of the effect of pulp-capping procedures reported the migration of dental pulp cells in the place of the formation of the reparative dentin and dentin bridge[48,54,55,56]. Naturally derived collagen or synthetic materials, such as polyglycolic acid (PGA), are used as a scaffold for attachment and guidance of cells[57]. The pulp-derived fibroblasts adhering to the PGA fibers can proliferate and form a new tissue similar to that of native pulp[58]. The synthetic matrices, however, must undergo degradation simultaneously with the new tissue formation by the cultured cells. The further investigation of DPSC should be focused on finding the appropriate biomaterials that would maximize the migration of these cells into the place of injury. Besides that, the key to successful tissue repair is also angiogenesis. DPSC express CD146 on their surface, the perivascular marker. It was shown that vascular endothelial growth factor (VEGF) and FGF promote neovascularization of severed human dental pulps[59]. These results could imply the possible application of angiogenic growth factors in dentin matrix[60] and their release after injury, which might be crucial for the local up-regulation of angiogenesis at injury sites.

## DENTAL STEM CELLS AND ADIPOGENESIS, CHONDROGENESIS, AND OSTEOGENESIS

Although the interest for DPSC arises mostly for their ability to differentiate into odontoblast-like cells, these cells are multipotent and have the possibility to differentiate into other cell types.

The cultivation of DPSC in potent adipogenic-inductive medium that contained isobutylmethylxanthine, hydrocortisone, and indomethacin induced DPSC to form characteristic oil red O-positive lipid-containing adipocytes[3,61]. This phenotypic conversion also correlated with the expression of the early adipogenic master gene PPARγ2 and the late marker lipoprotein lipase. Unlike BMDSC, DPSC failed to differentiate into adipocytes after treatment with glucocorticoids[4]. It is reported that only the $CD34^-$ subpopulation of dental pulp cells had the capacity to differentiate into adipocytes, odontoblast-like cells, and to form spheres comprised of cells expressing neural crest stem cell markers and of differentiating into Mart-1–positive melanocytes when grown in melanocyte-inducing medium[62].

DPSC also have the ability to differentiate into chondrocytes. The experiments with side population cells isolated from porcine dental pulp reported the expression of aggrecan and type II collagen mRNA in the culture of these cells, and that almost 30% of cultured dental pulp cells were converted into chondrocytes[21]. Stevens and coworkers showed that human DPSC after cultivation in chondrogenic medium express aggrecan and dental matrix protein-1 (DMP-1), which are both crucial for postnatal chondrogenesis[62,63]. The subcutaneous transplantation of DPSC to 1-day-old Swiss mice is followed by the generation of aggrecan- and Alcian blue–positive cartilage tissue in two out of three mice [62].

The ability of DPSC to produce chips of bone tissue as well as their high proliferative potential makes them good candidates for the study of bone formation[17,64]. DPSC have the ability to differentiate into osteoblasts and endothelial cells, and are capable of forming the woven bone[64]. The osteoblastic potential of these cells was proven by expression of RUNX-2, a transcription factor essential for inducing osteoblast differentiation[18,65] and the endothelial cells were confirmed by the expression of $CD54_b$, an antigen specifically expressed by endotheliocytes[64,66]. This synergy is most probably mediated by flk-1, which is shown to couple osteogenesis and vasculogenesis after transplantation of bone chips in immunosuppressed rats[18,67].

## DENTAL STEM CELLS AND NERVE CELLS

Neuronal stem cells have been isolated from various tissues, including brain, bone marrow, and retina[68,69,70]. The cultivation of these cells in the serum-free culture medium supplemented with epidermal growth factor (EGF) and bFGF leads to the formation of the cellular suspensions called neurospheres[68,71]. Neuronal stem cells have the ability to transform into neurons and glial cells, which is preceded by migration of the cells out of the spheres. Even more, the implantation of grafts of neural stem cells showed promising results as the potential therapy for various disorders of nervous system[72,73]. However, their accessibility and the potential risks for the patients that might occur during their extraction remain a problem.

The fact that dental pulp is derived from the neural crest and that it is shown that dental pulp cells show a high expression of nestin (marker for neural progenitors), nerve growth factor (NGF), brain-derived neurotrophic factor (BDNF), and glial cell line–derived neurotrophic factor (GDNF) mRNA transcripts opened the possibility of using dental pulp as the possible source for neural progenitors[74,75].

Dental pulp–derived cells promote the survival of sensory neurons of the trigeminal ganglion and of motoneurons of the spinal cord by providing neurotrophic support. Also, the increased survival of the motoneurons in the injured spinal cord after the implantation of the dental pulp cells grafts was reported[76]. NGF, BDNF, and GDNF produced by dental pulp cells promote the survival and neuroprotection of dopaminergic neurons against 6-hydroxy-dopamine *in vitro*[75]. Dental pulp grafted intraocularly led to the increase of density of catecholaminergic nerve fibers in the iris that is, probably, due to the presence of the neurotrophic factors in the anterior chamber of the eye[76,77].

Dental pulp cells from the adult rat incisor in culture have the ability to form neurospheres. The formation and growth of neurospheres is dependent on bFGF, but not on EGF, and is negatively regulated by TGF-β in a dose-dependent manner. Dental pulp–derived neurospheres show limited self-renewal ability[71]. DPSC induced by activators of the protein kinase C (PKC) and the cyclic adenosine monophosphate (cAMP) give rise to neuronal cells and, in smaller number, to glial cells, which are believed to be substantial for normal development and function of the neurons. These cells express neuronal and glial markers, and display voltage-dependent sodium and potassium currents[78].

Previous studies revealed that DPSC, when transplanted into adult rat/mouse brain, infiltrated into the host nerve tissue and expressed neurospecific markers[5,76]. Also, the transplantation of DPSC into the mesencephalon of a 2-day-old chicken embryo resulted in their differentiation into neuronal-like cells, with expression of neurospecific markers and the ability to produce the sodium currents[79]. It was shown that implantation of DPSC into the hippocampus of mice stimulates proliferation of endogenous neural cells and results in the recruitment of pre-existing nestin(+) neural progenitor cells (NPC) and βIII-tubulin(+) mature neurons to the site of the graft[80]. The injection of human neuronal stem cells isolated from the third molars into the dorsolateral striatum of rats inflicted with middle cerebral artery occlusion shows a significant recovery from neurologic dysfunction, which implies a possible therapeutic role of these cells in stroke-inflicted rats[81].

## DENTAL STEM CELLS AND TOOTH TISSUE ENGENEERING

Tissue engineering is a potential resolution for tissue/organ replacements and has been made possible by the cooperation of biological and material sciences. The concept involves the use of *ex vivo* expanded cells grown on a support of biocompatible material under appropriate environmental conditions in order to create tissue replacement and living prostheses[9].

Currently, there are two main branches of research of tooth tissue regeneration. The first deals with the restoration of partial tooth damages. This line of research focuses on using existing reparative capacities of the tooth and/or use of tooth-related stem cells for repair of damaged tooth parts. The second line of research focuses on whole tooth regeneration by using stem cells and conventional tissue engineering techniques[82]. The researches concerning the whole tooth regeneration developed into two main directions: (1) *in vitro* cell culture and polymers for *in vivo* implantation and (2) *in vivo* implantation of engineered cells[9]. However, the immune response of the human organism to the implants of dental stem cells is still unknown, and that question should be resolved before any clinical trials.

Now we know that it is possible to regenerate tooth crowns if the appropriate environment is provided, such as *in vitro* organ culture, grafts on chick chorioallantoic membrane, ocular grafts, subcutaneous transplants, or kidney capsules. The other important conditions are to create a three-dimensional (3D) organization of cells, to support the differentiation of these cells, and to avoid the xenograft rejection[9].

The attempts to make a bioroot also showed some promising results. The insertion of hydroxyapatite/tricalcium phosphate (HA/TCP) blocks containing SCAP and PDLSC into the socket of swine led to the regeneration of the root/PDL complex, over which an artificial dental crown could have been affixed. In this way, engineered bioroots showed lower compressive strength than that of natural swine root dentin, but they were capable of supporting a porcelain crown and resulted in normal functions[83].

The implantation of PDLSC seeded on HA/TCP in surgically created periodontal defects in miniature swine showed their excellent capacity to form bone, cementum, and PDL[84]. These results are promising, bearing in mind that periodontitis is one of the leading causes of tooth loss, especially in the elderly population[85].

A dentin pulp–like structure was obtained after the subcutaneous transplantation of DPSC/poly(lactic-co-glycolic) acid scaffold construct in New Zealand white rabbits. Transplanted samples revealed the formation of osteodentin-like structures, as well as tubular bilayered structures of vertically aligned parallel tubules resembling tubular-like dentin[86].

Toothlike structures can be created from biodegradable polymer scaffolds (collagen-coated polyglycolic acid, calcium phosphate material, collagen sponges) seeded with dissociated single-cell suspension obtained from pig, rat, or mice tooth germs and grown in the omentum of immunocompromised mice. The obtained structures contain enamel, dentin, and pulp, but are still missing some essential elements, such as the complete root and periodontal tissues that allow correct anchoring into the alveolar bone, and do not reach the expected size or shape of the scaffold[87,88,89].

Recently, a new approach was proposed for growing teeth in the mouse mandible[90]. In this study, epithelial and mesenchymal cells were sequentially seeded into a collagen gel drop and then implanted into the tooth cavity of adult mice. With this technique, the presence of all dental structures, such as odontoblasts, ameloblasts, dental pulp, blood vessels, crown, PDL, root, and alveolar bone, could be observed[90]. Cell-scaffold constructs in a coculture system appeared to improve the tooth shape control. PGA mesh and latter collagen sponge, together with the sequential seeding of dental mesenchyme in direct contact with dental epithelium, demonstrated organized tooth structures derived from dissociated postnatal canine and porcine molar tooth germs[91,92].

DPSC have been used for partial tooth structures, but not a whole biological tooth germ. Because of the limited *in vitro* expansion ability of DPSC, SHED, and other dental stem cells, efforts have been directed toward establishing cell lines; several lines have been created by the insertion of transgenes, including human telomerase reverse transcriptase, SV40 T antigen, and human papillomavirus genes, or by the spontaneously immortalization of dental follicle cells, cementoblasts, dental papilla, PDL, cervical loop epithelium, ameloblast, and odontoblast lineage cells[9]. One of the major technical advantages is that cells can be produced, characterized, and controlled relatively easily without the need to derive material repeatedly from primary tissue[9]. These lines could therefore be used for generating dental structures *in vivo*.

## ACKNOWLEDGMENTS

This work was supported by a grant, No 145004, from the Ministry of Science and Technological Development of Serbia. The authors declare no conflict of interests.
